# Groupwise Non-Rigid Registration with Deep Learning: An Affordable Solution Applied to 2D Cardiac Cine MRI Reconstruction

**DOI:** 10.3390/e22060687

**Published:** 2020-06-19

**Authors:** Elena Martín-González, Teresa Sevilla, Ana Revilla-Orodea, Pablo Casaseca-de-la-Higuera, Carlos Alberola-López

**Affiliations:** 1 Laboratorio de Procesado de Imagen, E.T.S.I. Telecomunicación, Universidad de Valladolid, Paseo Belén 15, 47011 Valladolid, Spain; casaseca@lpi.tel.uva.es (P.C.-d.-l.-H.); caralb@tel.uva.es (C.A.-L.); 2 Unidad de Imagen Cardiaca, Hospital Clínico Universitario de Valladolid, CIBER de Enfermedades Cardiovasculares (CIBERCV), 47005 Valladolid, Spain; tereseru@gmail.com (T.S.); arevillao@saludcastillayleon.es (A.R.-O.)

**Keywords:** non-rigid registration, elastic motion, CNN, deep learning

## Abstract

Groupwise image (GW) registration is customarily used for subsequent processing in medical imaging. However, it is computationally expensive due to repeated calculation of transformations and gradients. In this paper, we propose a deep learning (DL) architecture that achieves GW elastic registration of a 2D dynamic sequence on an affordable average GPU. Our solution, referred to as dGW, is a simplified version of the well-known U-net. In our GW solution, the image that the other images are registered to, referred to in the paper as *template image*, is iteratively obtained together with the registered images. Design and evaluation have been carried out using 2D cine cardiac MR slices from 2 databases respectively consisting of 89 and 41 subjects. The first database was used for training and validation with 66.6–33.3% split. The second one was used for validation (50%) and testing (50%). Additional network hyperparameters, which are—in essence—those that control the transformation smoothness degree, are obtained by means of a forward selection procedure. Our results show a 9-fold runtime reduction with respect to an optimization-based implementation; in addition, making use of the well-known structural similarity (SSIM) index we have obtained significative differences with dGW with respect to an alternative DL solution based on Voxelmorph.

## 1. Introduction

Image registration is the process of aligning two or more images for subsequent joint processing; it is especially widespread in the medical imaging field either for processing images acquired in different time instants—or with different imaging devices—for the same patient or to carry out comparative analyses with multiple patients [1]. Registration is a three-tier process, in which (a) a deformation model, (b) a measure of similarity between the entities to be aligned and (c) an optimization procedure, must be defined. Rigid models are often used for brain longitudinal studies on the same patient, while elastic models are used for moving deformable organs, such as the heart. Optimization procedures must be chosen according to the deformation model employed.

According to the deformation model, registration may be labelled as rigid or non-rigid; the latter is still subdivided as affine and elastic. Elastic motion applies for natural deformable structures such as a beating heart. As for the measure of similarity, both monomodal and multimodal metrics are needed; monomodal metrics come into play when images are obtained with the same imaging process (same medical modality or same frequency); otherwise, multimodal metrics are mandatory. Registration of monomodal dynamic medical images is frequently encountered because of patient motion, either involuntary or spontaneous (heart pumping or breathing) as well as small voluntary motion due to discomfort during the scanning or to non-cooperative patients (children, elderly…). Follow up of patients is also an application domain for this type of registration [2].

Registration of multiple images can be tackled as a pairwise (PW) or as a groupwise (GW) problem. The former may be carried out sequentially, i.e., the next image in the sequence is registered against the previous image or, alternatively, one of the images in the sequence may be selected as the reference image while the others will be registered (in pairs) against that reference. On the other hand, the GW approach consists of a joint optimization problem in which the reference is created with information of the whole temporal sequence. GW methods typically outperform PW since the problem is solved as a whole. However, this comes at the expense of a higher computational cost, due to their iterative nature [3].

In this paper, we focus on providing a deep learning (DL)-based elastic GW registration solution with respect to an optimization-based traditional solution. We propose a much simpler network than that described in [4] (see Section 2 for more insight on references in this paragraph); our network fits a single affordable GPU within an average workstation to solve a GW elastic and dynamic registration problem; in addition results will show that our solution outperforms the increasingly popular Voxelmorph [5,6]. The application we have in mind is our previously reported fast motion-compensated 2D-cardiac cine MRI reconstruction [7] and, specifically, to replace the ME/MC stage there described with a DL-based solution. In this paper, we include, for completeness, a reconstruction comparative with both results; we show that computational costs are greatly diminished while keeping an acceptable performance.

## 2. Related Work

The field of registration has a long record [8]; recently the DL paradigm has entered the field with solutions that provide fast registrations once networks have been trained. On the one hand, supervised solutions such as [9,10] rely on segmentations or landmarks to estimate the displacements. On the other hand, there are unsupervised solutions such as [11,12] that do not require any ground truth. The Voxelmorph learning framework [5,6] belongs to the second group and parametrizes deformations using a convolutional neural network (CNN) to implement a PW solution. In the GW arena, we are aware of three attempts that make use of DL technology. In [4], the authors use a very involved architecture with a large number of parameters as well as several skip connections; it is applied to the registration of multimodal static ocular images. In [13,14], the registration is applied to brain MR scans. While in the first case supervised learning is used, in the second case deforming autoencoders are employed. None of these three alternatives apply GW registration to dynamic sequences.

2D Breath-hold (BH) cardiac cine magnetic resonance imaging (MRI) is the most common clinical protocol employed to assess both the anatomy and the function of the heart. This modality is known to suffer from some drawbacks so different alternatives have been proposed, with 3D free breathing (FB) being rendered as the natural extension. However, regardless of the dimensions or the BH/FB property, since acquisition times remain long as compared with other modalities, there is a pressing need for faster solutions. A tremendous effort has been carried out in overcoming this issue using strategies such as aggressive undersampling, fancy k-space trajectories or powerful optimization algorithms [15] where exploiting redundancy reveals itself as the cornerstone. To this end, reconstruction methods based on motion estimation and compensation (ME/MC) have been reported, which make use of both PW [16] and GW [7,17] solutions. Recently, DL solutions have also emerged in the field of dynamic image reconstruction, where the optimization problem is unrolled. These types of solutions aim at an overall speed-up as well as eliminating the need for hyperparameter tuning [18]. Additional efforts have been reported with recurrent neural networks to avoid the need for unrolling the optimization process [19]. However, a combined approach with DL and traditional optimization has been recently described [20]; such a combination puts together the power of data as well as the previous knowledge of the forward models that may apply in a particular application domain, such as the MR reconstruction problem.

## 3. Material and Methods

### 3.1. Materials

We have used two databases of 2D cardiac cine MRI with short axis orientation. The first database consists of multi-slice acquisitions from 89 subjects, both healthy and affected by hypertrophic cardiomyopathy (in the proportion 1/3 and 2/3, respectively). This database has been previously used by our team elsewhere [21,22]. This database will be hereinafter referred to as Database I.

The different slices have been extracted from each subject as independent 2D dynamic sequences. The dataset has been separated into two subsets, namely training and validation, containing 609 and 151 two-dimensional sequences, respectively. Each of the dynamic sequences contains N=30 time frames—Notice that the cardiac cycle is divided into *N* equally spaced cardiac phases so separation between frames is patient-dependent. In addition, arrhythmia episodes, provided they occur during the acquisition, are automatically discarded by the MR equipment—The images were pre-processed so that they had a 1 ${\mathrm{mm}}^{2}$ resolution, and then cropped to 320 × 320 pixels. Finally, each of the dynamic sequences was intensity-normalized between 0 and 1.

The second database was used for validation and testing. It consists of 41 subjects with at least an ischemia episode. In this case, acquisitions were single-slice, in which the slice was relatively centered in the base-apex direction; the number of frames was also equal to 30. Images had different in plane resolutions but, as before, they all were pre-processed so that they had a 1 ${\mathrm{mm}}^{2}$ resolution, and then cropped to 320 × 320 pixels. Images in the sequences were also intensity-normalized between 0 and 1. This database will be hereinafter referred to as Database II.

### 3.2. Reconstruction Problem

Cardiac cine MR images can be reconstructed with high quality from highly undersampled data. The reconstruction algorithm we use was published in [7] and later improved in [23]. It departs from the multi-coil k-space subsampled information and solves a compressed sensing reconstruction problem. This is done following a classical optimization procedure based on a data fidelity term and a regularization term. In this second term, sparsity is fostered by means of penalizing the $\ell_{1}$ norm of the temporal total variation of the registered image set; this is where the GW-MC operator is applied [7]. Specifically, the reconstruction problem is defined by:

(1) $$\min_{m}\frac{1}{2}{∥y-Em∥}_{\ell_{2}}^{2}+\lambda {∥\Phi Tm∥}_{\ell_{1}}$$

where Φ is the sparsifying transform, T is a groupwise MC operator that includes transformations as well as interpolations, *m* is the dynamic MRI image set to be reconstructed and *y* is the undersampled multi-coil k-space data, respectively. The encoding operator *E* performs a frame-by-frame undersampled spatial Fourier transform and includes the multiplication by coil sensitivities [23].

The reconstruction process is iterative, so that with each iteration the provided solution is refined. This means that registration must be performed several times. The registration itself is also iterative, since transformations and gradients therein must be repeatedly calculated. This is highly computationally expensive, so registration constitutes the bottleneck of the reconstruction algorithm. To reduce that load, in this paper we replace the original registration algorithm used to implement operator T in Equation (1) with a DL-based solution, say $T_{DL}$, keeping the rest of the reconstruction algorithm unaltered.

### 3.3. Registration

As stated in the Introduction, the purpose of image registration is to transform the images so that corresponding structures coincide. In the cardiac reconstruction problem, elastic deformations are applied to the original sequence so that the registered sequence looks static; ideally, the registered frames in the time sequence should all be pixelwise equal, so the total variation term would be null. Following [7], an elastic deformation is achieved by means of free-form deformations [24], using as a metric the sum of squared differences (SSD) between the images in the sequence and the reference (the reference would be hereinafter referred to as *template*); the GW approach consists of solving a joint problem to find out both the optimum transformations of the images in the time sequence to the template, as well as the calculation of the template itself. This decision is made to avoid the bias that would stem from choosing beforehand one of the images in the sequence as the template [25]. For SSD, the optimum template is known to be the average of the registered images [1]. Hence, the SSD is defined as

(2) $$SSD\left(T\left(x\right)\right)=\frac{1}{N}\sum_{n=1}^{N}{\left(m_{n}\left(T_{n}\left(x\right)\right)-\frac{1}{N}\sum_{n^{\prime }=1}^{N}m_{n^{\prime }}\left(T_{n^{\prime }}\left(x\right)\right)\right)}^{2}$$

where $T=\{T_{1},\ldots ,T_{N}\}$ is the set of transformations, *N* is the number of frames in the sequence, $m_{n}$ the *n*-th frame and $T_{n}$ is the transformation that maps each material point *x* in the template onto its corresponding position in the *n*-th frame.

Overall, the registration problem is posed as

(3) $$\min_{T}\sum_{x\in X}SSD\left(T\left(x\right)\right)+F\left(T\left(x\right)\right)$$

where F(T(x)) is a function that imposes some regularity conditions or constraints on the transformations and X is some region in the template domain; this region could include the whole template or could be limited to some parts of the template (for instance, the cardiac area). Equations (2) and (3) show that registration needs the reconstructed images, which are obtained by minimizing (1) which, in turn, requires the registration solution. Therefore, as previously stated, the problem is iterative in nature.

### 3.4. Registration via Deep Learning

**Figure 1:**
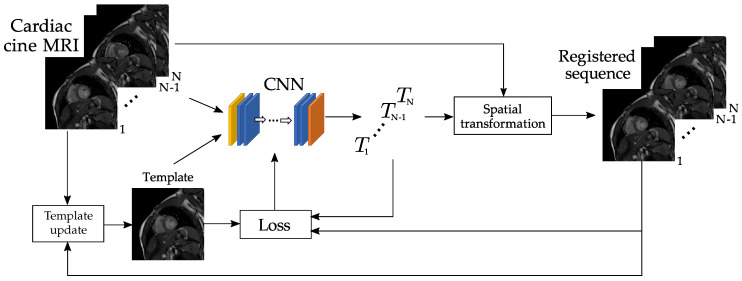
Outline of the proposed dGW registration network. A 2D cardiac cine sequence consisting of *N* frames enters the network, one at a time together with the template, which is calculated as described in Section 3.4.2. The output of the *N* executions of the CNN is a sequence of *N* 2D deformation fields. The original frames, together with their two-channel deformation fields, enter the *Spatial transformation* block, giving rise to the registered images. This process is applied L+1 times (see Section 3.4.4), both for training and for prediction.

In this paper, we solve the problem posed in Equations (2) and (3) by means of a DL-based approach. The pipeline, hereinafter referred to as deep groupwise (dGW), is depicted in Figure 1; the solution consists of a CNN that is sequentially fed with each of the images in the time sequence to be registered together with the available template. The network provides a sequence of the deformation fields (i.e., transformations $T_{n}\left(x\right),\forall x\in X,n=\left\{1,\ldots ,N\right\}$) and it is followed by a spatial transformation module that provides the registered images.

The solution proposed is iterative; specifically, we refer to an iteration as the set of actions that begins by feeding the network with the first frame in the data sequence and finishes with the output of the last frame in the sequence. The final solution is obtained after several iterations; this is the first hyperparameter that has been set in the design by means of training and validation. In the first iteration, i.e., when no output is available yet, the template is one of the input frames, which is selected to minimize a geodesic distance criterion; details are provided in Section 3.4.2. In subsequent iterations, the template is updated by averaging the transformed images from the previous iteration. Let *L* be the number of template averages performed; the number of iterations will be denoted by L+1.

The main ingredients in the figure are now described in detail.

#### 3.4.1. Architecture

**Figure 2:**
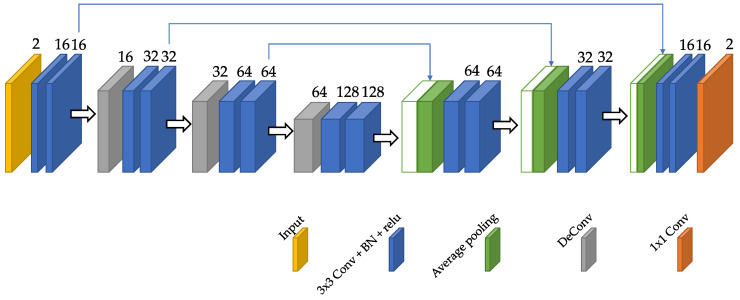
Proposed convolutional architecture of the dGW network. The number of channels is shown above each layer. Skip connections involve concatenation of feature maps extracted in the encoding stage with new feature maps from the decoding stage.

We propose a CNN based on the well-known U-net [26], widely used in medical imaging. Figure 2 shows the network architecture; the network has some resemblance with the network proposed in [4] although we have reduced substantially the number of filters so that it fits an average GPU. As can be inferred from the figure, both input and output have two channels. With respect to the former, each channel corresponds to a particular image frame and the available template. As for the output, each channel represents the displacement of each material point in one coordinate direction. The figure also shows each constituent block, the details of which are coded both with a color and a legend. Numbers on top of each block in the network indicate the number of channels. Skip connections are also included both to maintain information from previous layers, as well as to avoid the problem of vanishing gradients during backpropagation.

#### 3.4.2. Template Update

As previously stated, the *Template update* block carries out two different operations, depending on the iteration number. For iterations l≥1 this module performs a pointwise average of the registered images due to its optimality for SSD [1]. However, any other template construction policy could be accomplished for other types of similarity measures [8].

As for the initial iteration (l=0), we resort to the selection of the template by minimizing the geodesic distance of images to the template on an empirical manifold constructed by means of a k-nearest neighbor (kNN) graph [27]. Specifically, the steps to find the reference using this method are the following [21]:

(a) Define the similarity between two frames as the residual complexity [27].
(b) Construct the connected kNN graph of frames based on that similarity, where k is the number of neighbors of each frame. A frame is considered connected to its k-closest frames, the higher k, the more connected the graph.
(c) Compute the geodesic distance of every node in the graph.
(d) Select the node, i.e., the frame, with a minimum sum of distances to the rest of the frames. This frame will be taken as the template.

Hereinafter we will refer to this procedure as *automatic template selection*. The template enters the network together with the dynamic sequence, obtaining at its output the deformation fields used by the Spatial Transformation block to give rise to the registered sequence. This registered sequence will be used for the calculation of the template in the next iteration.

#### 3.4.3. Loss Function

The proposed network may be trained by minimizing any derivable loss function; hence, despite in this paper we focus on a monomodal application domain, the network lends itself to both monomodal and multimodal registrations by appropriately choosing the similarity function in Equation (4). Specifically, in our case the loss function is:

(4) L(T(x))=Similarity(T(x))+Regularization(T(x))+Constraint(T(x))

As for the similarity metric, we focus on SSD (see Equation (2)) for the monomodal case [7]. The network has also been tested with *cross-correlation* (CC), defined as follows:

(5) $$\begin{array}{cc} CC(T(x)) & =\sum_{x\in X}\frac{{\left(\sum_{x_{i}}\left(f\left(x_{i}\right)-\hat{f}\left(x\right)\right)(m\left(T\left(x_{i}\right)\right)-\hat{m}\left(T\left(x\right)\right)\right)}^{2}}{\left(\sum_{x_{i}}{\left(f\left(x_{i}\right)-\hat{f}\left(x\right)\right)}^{2}\right)\left(\sum_{x_{i}}\left(m\left(T\left(x_{i}\right)\right)-\hat{m}\left(T\left(x\right)\right)\right){)}^{2}\right)} \end{array}$$

with f(x) and m(T(x)) are the template and the deformed images, respectively; $\hat{f}\left(x\right)$ and $\hat{m}\left(T\left(x\right)\right)$ denote local mean intensity images. $x_{i}$ iterates over a square region around *x* with a 9-pixel size in each dimension. Notice that the better the alignment the higher CC; additionally, this function always provide values between zero a one; consequently, the loss function uses the negative of CC, hereinafter, NCC.

In addition, since smooth deformation fields are expected, function F(·) in Equation (3) comprises some regularization terms in the first and second-order spatial and temporal gradients; specifically:

(6) $$Regularization\left(T\left(x\right)\right)=\sum_{x\in X}\sum_{n=1}^{N}\sum_{p=1}^{4}\lambda_{p}R_{p}\left(T_{n}\left(x\right)\right)$$

with X as defined in Equation (3). $\lambda_{p}$ are the weights of the regularization terms $R_{p}$, which are defined as:

(7) $$\begin{array}{cc} R_{1}\left(T_{n}\left(x\right)\right) & ={\left|\frac{\partial T_{n}\left(x\right)}{\partial x_{1}}\right|}^{2}+{\left|\frac{\partial T_{n}\left(x\right)}{\partial x_{2}}\right|}^{2} \end{array}$$

(8) $$\begin{array}{cc} R_{2}\left(T_{n}\left(x\right)\right) & ={\left|\frac{\partial^{2}T_{n}\left(x\right)}{\partial x_{1}^{2}}\right|}^{2}+{\left|\frac{\partial^{2}T_{n}\left(x\right)}{\partial x_{2}^{2}}\right|}^{2}+2{\left|\frac{\partial^{2}T_{n}\left(x\right)}{\partial x_{1}\partial x_{2}}\right|}^{2} \end{array}$$

(9) $$\begin{array}{cc} R_{p}\left(T_{n}\left(x\right)\right) & ={\left|\frac{\partial^{q}T_{n}\left(x\right)}{\partial n^{q}}\right|}^{2},\;q=p-2,\;p=\left\{3,4\right\}, \end{array}$$

with $x=(x_{1},x_{2})$ the two coordinates of material point *x*. With respect to the temporal derivatives in Equation (9) we have used, in an abuse of notation but for simplicity, the time index *n* as a continuous variable. All the derivatives are approximated by finite differences.

Function F(·) in Equation (3) also comprises the penalty described in [28], intended to constrain the space of solutions. Since the time sequences under study comprise a cardiac cycle due to a synchronized acquisition with the ECG signal, periodicity is assumed, which is enforced by the constraint that the sum of the displacements from a given material point is zero. This can be written as:

(10) $$Constraint\left(x\right)=\lambda_{c}\sum_{x\in X}{\left(\frac{1}{N}\sum_{n}\left(T_{n}\left(x\right)-x\right)\right)}^{2}$$

#### 3.4.4. Training

**Figure 3:**
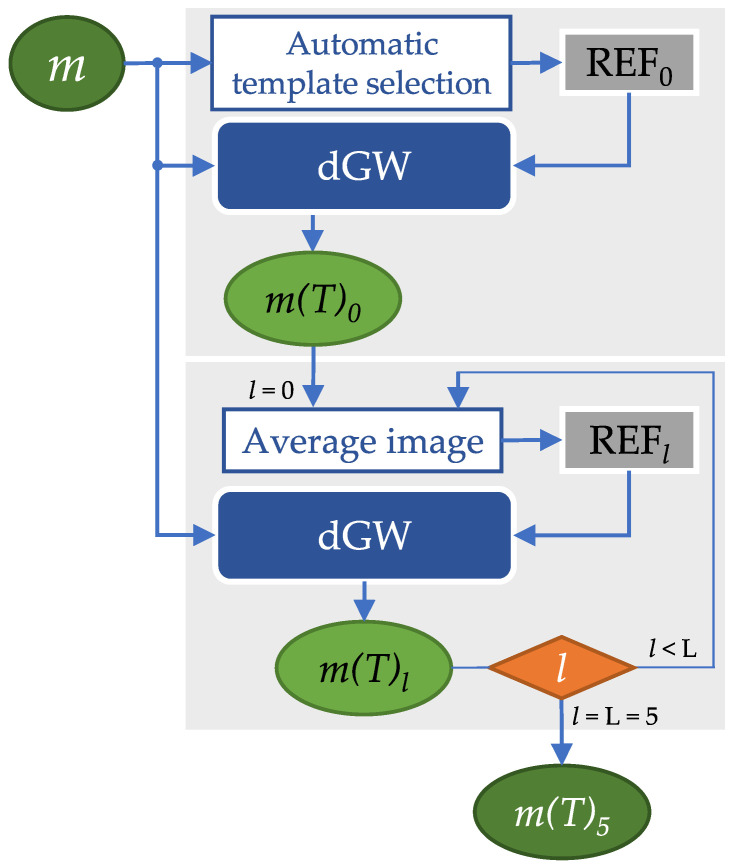
dGW registration pipeline. The original 2D cardiac cine MRI sequence *m* enters both the network and the template selection block (see Section 3.4.2, l=0). The output is the registered sequence $m{\left(T\right)}_{0}$, which is used to calculate the template for l=1. This is cycled until l≤L=5.

The proposed training scheme is now explained (see Figure 3 for reference). Given an epoch, i.e., one passage of the whole training set through the network for parameter update, the order in which dynamic sequences enter the network is randomly chosen, so that sequences belonging to the same subject do not enter consecutively; then, for each sequence, the steps taken are:

1. Set l=0. The template is calculated as described in Section 3.4.2.
2. The batch size is taken as the number of frames per slice; therefore, following the definition of *iteration* given in Section 3.4, network parameters are updated at the end of each iteration; at this moment, the registered sequence $m{\left(T\right)}_{0}$ is obtained.
3. Set l=l+1. Update the template as the average of the registered sequence at the previous iteration.
4. Steps 2 and 3 are executed while l≤L=5; consequently, 6 templates are calculated for a given slice. The output at this stage is considered to be the final registration output. The number L=5 is a parameter design that has been set beforehand.

The overall training consists of ten epochs; images employed for testing were those from Database II, as described in Section 3.1.

When dGW is used in prediction mode, the steps taken are the same as for training. As is customary, the selection of hyperparameters of the trained networks is carried out using the results on the validation set. Final network performance is calculated on the test set.

#### 3.4.5. Implementation

This proposed network is implemented in Tensorflow and Keras [29,30]. The Adam optimizer [31] was used to train the network, with a learning rate of $10^{-4}$. We also use *neuron* [32] to carry out spatial transformations. Tensorflow function add_loss has been used to accommodate the three terms defined in Equation (4), which have been written as different functions.

**Figure 4:**
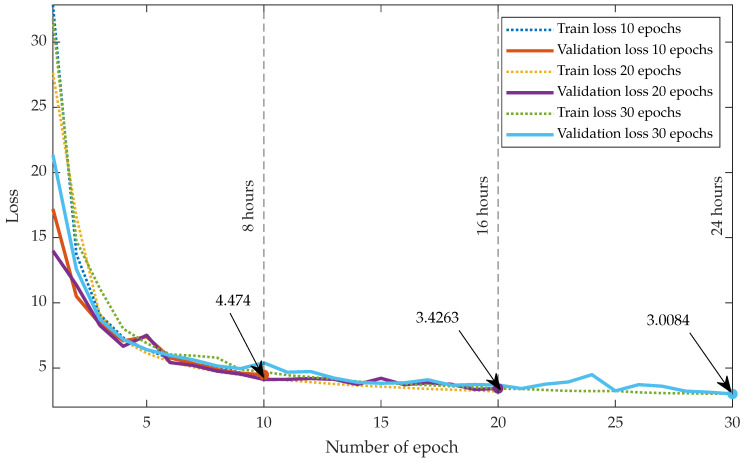
Loss evolution during training.

Each network training for 10 epochs and using a batch size of 30—which corresponds, as previously stated, with the number of frames—took 8 h on one Intel^®^ Core™ i7-4790 CPU @ 3.60 GHz with 16 GB RAM and one NVIDIA GeForce RTX 2080 Ti GPU. This equipment is deemed as *affordable* since it is quite common in an average research lab. Figure 4 shows a graphical representation of the loss function on both the training and the validation datasets from Database I as the number of epochs. Choosing several epochs equal to 10 seems an adequate trade-off between performance and runtime.

### 3.5. Performance Analysis and Hyperparameter Selection

We evaluated registration performance using the SSIM index [33]; this measure incorporates three sources of information to measure similarity between two images, namely luminance, contrast and structure, where the latter is quantified as a measure of cross-correlation. In our problem, because the ideal registration should give rise to a registered dynamic sequence perfectly indistinguishable from the template, SSIM is a suitable measure to evaluate the registration quality. For each dynamic sequence in the validation/test dataset two distant frames are selected to build a sample of SSIM values for a particular algorithm; using all the frames in the sequence would cause correlation within the sample to grow. These frames correspond to systole and diastole. Comparative performance analysis is carried out on the basis of the sample of SSIM values from each algorithm that enters the comparison; boxplots will be shown, and paired unilateral *t*-tests *p*-values will be reported when necessary. Sign-tests have also been calculated to avoid non-normality issues. Unless otherwise stated *p*-values from *t*-tests will those reported; sign-test values will only be reported when *p*-values are in the vicinity of 0.05.

**Figure 5:**
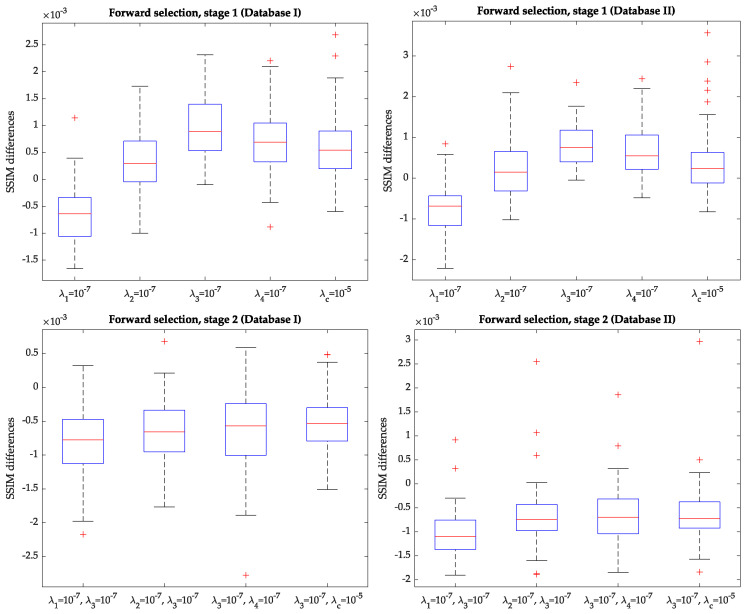
Differences between the SSIM samples for the validation sets in Database I (**left**) and Database II (**right**). (**Upper row**) Pairwise differences in Stage 1 with each parameter taking a non-null value and the SSIM sample obtained from the network in Stage 0. (**Lower row**) Pairwise differences in Stage 2 for the best selection of each parameter within the pair indicated above.

The five hyperparameters to select are those in Equations (6) and (10). To sample the parameter space, we resorted to a *forward selection* procedure typically used in pattern recognition [34] for feature selection; being exhaustive in our problem would lead to large training and validation times, which we meant to avoid. Specifically, the process consists of several stages:

- Stage zero: all the parameters in Equations (6) and (10) are set to zero; the network is trained and the SSIM sample is obtained on the validation dataset.
- Stage one: we pursue to find the optimum value of each hyperparameter while the others are null. Then, the best among them is selected. For the four parameters in Equation (6), we test the values $\left\{10^{-7},5\times 10^{-7}\right\}$; for $\lambda_{c}$ in Equation (10) we tried $\left\{10^{-7},10^{-5}\right\}$; these values were preselected on the basis of the validation set in Database I. Then, for each hyperparameter and each value within the pairs just mentioned, we train a network and then a SSIM sample is obtained with each validation dataset. Notice that 2×5=10 networks are trained. SSIM values turned out to be quite close to the all-null solution indicated in Stage 0. Thus, we have obtained the boxplots of the pairwise differences between the SSIM sample with each parameter taking a non-null value and the SSIM sample obtained from the network in Stage 0. Figure 5, upper row, shows these boxplots for the validation sets in Database I (left) and Database II (right) for the best selection of each parameter within the pair indicated above (i.e., for the value of this parameter that provides the most favorable result to this parameter); clearly, $\lambda_{1}$ turns out not to be relevant, while the others provide positive-shifted distributions; in the four rightmost boxplots differences turned out to be significant with *p*-values $<10^{-4}$. As can be appraised, the highest median corresponds to the activation of $\lambda_{3}=10^{-7}$; this is observed with the two databases. Hence, the result of this stage is the setting of the first order (p=3) temporal derivative defined in Equation (7) to the value $10^{-7}$.
- Stage two: the purpose is to find whether the combination of two non-null hyperparameters (being one of them $\lambda_{3}=10^{-7}$) provides significantly different results than those provided by the network with $\lambda_{3}=10^{-7}$ and the rest of them null. To this end, new networks are trained with $\lambda_{3}$ fixed while the others can take values within the pairs indicated above. Then, 2×5=8 networks are trained and, for each one, a SSIM sample is obtained with each validation dataset. Should significant differences be found, the second hyperparameter would be that value associated with the minimum *p*-value. Figure 5, lower row, shows the corresponding pairwise differences in this stage for the best selection of each parameter within the pair indicated above for the validation sets in Database I (left) and Database II (right); the figure clearly shows that adding a second parameter does not provide better results than keeping $\lambda_{3}$ activated on its own since differences are negative. Therefore, the forward selection procedure ends at this stage, with the selection of a network with only one hyperparameter activated.
- Further stages: in the case that two parameters had been selected, this process would continue by setting a third parameter, with the selected two parameters from stage two remaining fixed, and would continue until all the parameters were set or no significant differences were obtained. Since such differences were not found on stage two, no further stages were tested.

## 4. Experiments

In this section, we provide an overview of the experiments we have conducted; when appropriate, we describe methodological details not directly related to our proposal to make the paper self-contained. Unless otherwise stated, all the comparisons will be based on the SSIM distributions of the solutions tested on the test set from Database II. Experimental results themselves are described in Section 5.

### 4.1. Experiment 1: Iterations, Time Sequence Ordering and an Alternative Similarity Metric

Once the network model has been selected and hyperparameters are set, we meant to test whether our iterative solution i.e., L=5, (recall Section 3.4.4 and Figure 3) was worth taking as opposed to providing a feedforward solution (L=0). To this end, the SSIM distribution of the outputs at l=0 and at l=L=5 have been compared.

For visualization purposes, we also show some images that result from the registration. In this case, we employ the Matlab function imshowpair where differences between the images are highlighted by means of pseudocolor; the presence of color means differences between the images with higher intensity for higher differences; green reddish tones can be appraised, depending on which of the two images shows a higher value at a particular pixel. Gray scale is maintained when pixel values are alike. Therefore, the more colored the pixels and higher the intensity in the representation, the higher dissimilarity between the images.

In addition, we also tested whether the order in which the frames enter the network make a difference. To this end, two experiments were conducted. In one of them, the first frame in the sequence was randomly chosen and then the time ordering was followed (when the end of the sequence was reached, we followed its periodic extension, i.e., the *N*-th frame was followed by frame number 1). The second experiments consisted a in random permutation of the frames within the dynamic sequence.

Finally, although we have focused on SSD, we have conducted an experiment to verify that our architecture can adapt to other types of similarity metrics in the loss function. To this end, we have used the NCC, defined in Equation (5); this function is calculated for each image in the sequence, i.e., 1≤n≤N, and its values are accumulated. Template selection and batch size remain as described in Section 3.4.4 and Section 3.4.5 and hyperparameters have all been set to zero. As for the SSD, we have tested both the network with all hyperparameters null as well as the solution we proposal as optimal, i.e., that with $\lambda_{3}=10^{-7}$.

### 4.2. Experiment 2: Performance Comparison with Another DL Architecture

The performance of our model was compared with two different alternatives of Voxelmorph (VM), namely one of the initial unsupervised learning variants (VM-2, as referred to in [5]) and the probabilistic and diffeomorphic formulation (VMdiff) [6].

The two VM implementations use two well-known metrics, namely mean square error a negative cross-correlation. The latter has been defined in Equation (5); as for the former, it is defined as:

(11) $$\begin{array}{cc} MSE(T(x)) & =\frac{1}{\left|X\right|}\sum_{x\in X}{\left(f(x)-m(T(x))\right)}^{2} \end{array}$$

with f(x) and m(T(x)) defined in Section 3.4.3.

VM networks were trained in two different ways as follows: (a) For each slice in the training set, the first image in the sequence is chosen as the template. The network parameters are updated once the remaining frames in the sequence are registered, i.e., batch size equals N−1. Then, the next frame is chosen as the template and the same process is repeated until all of the frames have acted as template; then, we move on to the next slice and continue until the epoch finishes; (b) For each slice in the training set the template is automatically chosen (following the procedure described in Section 3.4.2 for l=0), so batch size equals *N* as in dGW training; then, we move on to the next slice and continue until the epoch finishes

In prediction mode, the network operates as indicated in (b), i.e., the template is automatically chosen and the N−1 remaining frames are registered by means of the network.

Comparisons, in this case, are made on the basis of both SSIM as well as on other popular metrics, such as signal to error ratio (SER), Mutual Information (MI) [35] and Cross-Correlation [36]. We also show exampled images in a pseudocolor composition.

### 4.3. Experiment 3: dGW vs. an Optimization-Based Registration Approach

dWG has been compared with the optimization-based groupwise registration method described in [7] for ME/MC within an image reconstruction procedure. In this experiment the registration procedure is compared to our DL solution with fully sampled data. In this case, we use the same parameters as those mentioned in Section 4.2 as well as the same similar pseudocolor representation of the registered images.

### 4.4. Experiment 4: Dynamic Image Reconstruction

As a proof of concept, several reconstructions were performed using the GW method published in [7]; this method makes use of an ME/MC procedure using the groupwise optimization-based registration method mentioned in Section 4.3. dGW enters this field as a DL registration alternative to the original optimization-based procedure. The method in [7] is doubly iterative, i.e., it iterates in Equation (1) as well as to solve the registration problem, i.e., it also iterates in Equation (6). For simplicity, only one iteration in Equation (1) has been carried out, both for GW and dGW.

The experiments were conducted as follows: for a time sequence, each frame underwent a Fourier transform. Then, a fraction of the horizontal lines in the transform was retained while the rest were set to zero. The ratio between the lines retained and the overall number of lines is known as acceleration factor (AF). This operation is modelled by operator *E* in Equation (1). The experiments have been conducted with different AFs.

## 5. Results

### 5.1. Results of Experiment 1

**Figure 6:**
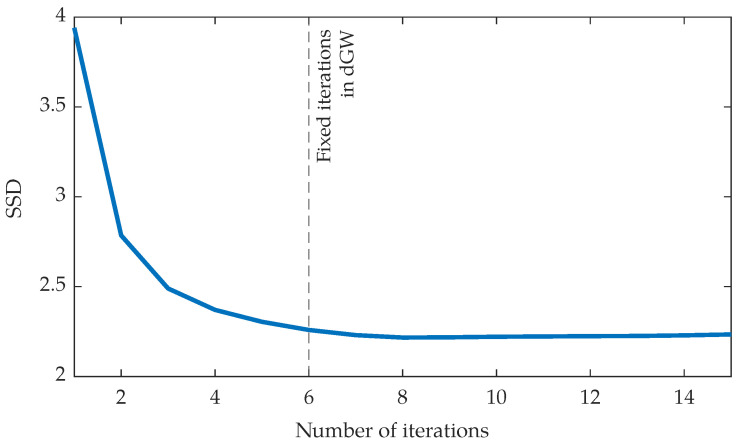
SSD obtained in dGW registration according to the number of iterations.

**Figure 7:**
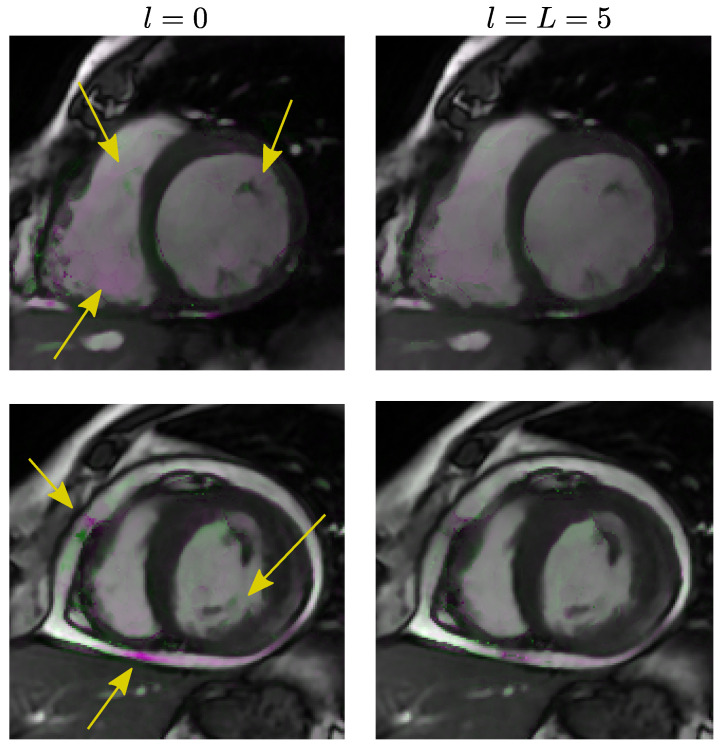
Differences between l=0 and l=L=5 in dGW registration quality. We show systole and a diastole superimposed frames for two slices in Database II. Leftmost column: dGW registration with l=0. Rightmost column: dGW registration with l=L=5. Pink: registered systole with higher intensity. Green: registered diastole with higher intensity.

As for the importance of having L=5 as opposed to L=0, i.e., an iterative vs. a feedforward solution, a paired *t*-test was conducted on the outputs obtained from the test data from Database II. The *p*-value was <$10^{-7}$, i.e., significant differences existed using L=5 as opposed to L=0. Figure 6 shows the evolution of SSD as a function of the number of iterations; we have drawn a vertical line of the number of iterations we set as a design parameter. As can be observed, the SSD value diminishes with the number of iterations but it gets stable around six. Figure 7 shows an example of two registrations with no iterations (left) and with six iterations (L=5, right); the SSD for the case L=0 are 5.65 (upper figure) and 8.67 (lower figure) while for the L=5 case the values are 2.49 (upper figure) and 4.52 (lower figure). Although differences are subtle, one can observe a general higher intensity in the color on the leftmost images; differences have been highlighted by means of arrows.

As for the importance of the ordering of the data frames within the dynamic sequence, the random selection of the first frame in the sequence gave rise to non-significant differences (p=0.05, sign-test p=0.0442). However, the random permutation did give rise to significant differences ($p=2.05\cdot 10^{-4}$). Consequently, no differences exist with a random selection of the cardiac cycle start but differences do exist when the time frames are randomly shuffled.

**Figure 8:**
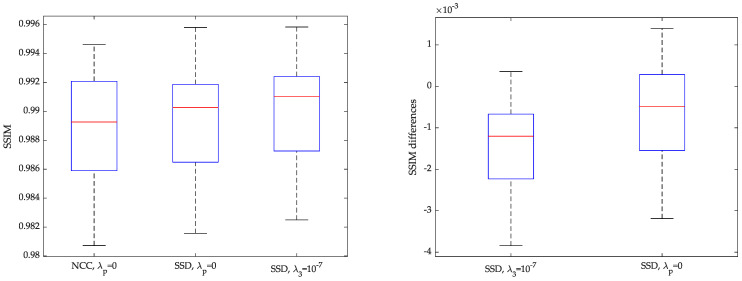
(**Left**) Distribution of SSIM samples in dGW registration, using NCC as similarity metric, SSD with null hyperparameters and SSD with $\lambda_{3}=10^{-7}$. (**Right**) Differences between the SSIM samples on the left.

**Figure 9:**
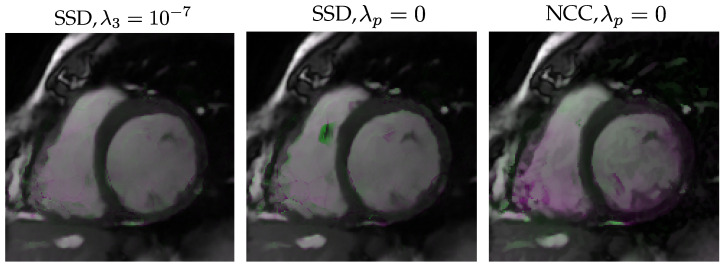
Differences between the implementation with NCC as a similarity metric and two models with SSD in dGW registration quality. We show systole and a diastole superimposed frames for a slice of a patient in Database II. From left to right: dGW registration with SSD and $\lambda_{3}=10^{-7}$, dGW registration with SSD and all hyperparameters null, dGW registration with NCC and $\lambda_{3}=10^{-7}$. Pink: registered systole with higher intensity. Green: registered diastole with higher intensity.

As for using NCC as opposed to SSD, Figure 8 shows boxplots of the solutions; specifically the leftmost figure shows SSIM, from left to right, values of the SSIM distribution for NCC, SSD with all parameters null and SSD with only $\lambda_{3}$ non-null. Distributions show some degree of overlapping. Pairwise differences are shown in the rightmost figure, where the first boxplot shows the case NCC— $\lambda_{3}=10^{-7}$ case and the second one shows case of NCC and all-null parameters for SSD. Differences in paired comparisons do exist $\left(p<10^{-7}\right)$ for both. Some example images are shown in Figure 9.

### 5.2. Results of Experiment 2

**Figure 10:**
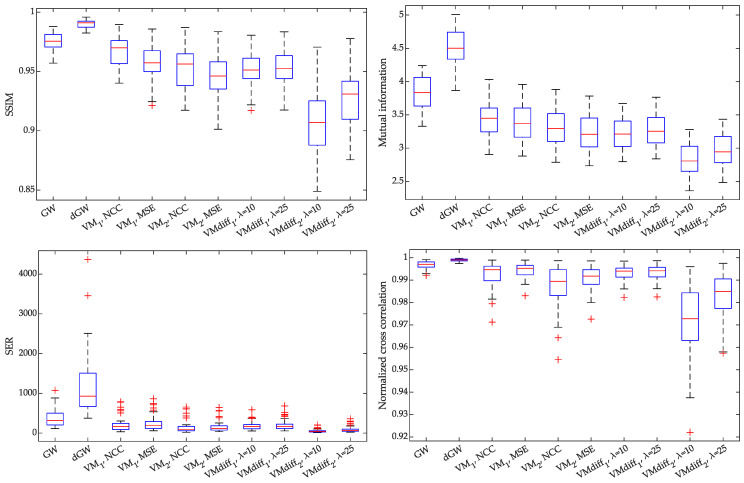
Performance comparison between dGW and other alternatives. Four different similarity metrics are used. Upper line: SSIM (**left**) and Mutual information (**right**); Lower line: SER (**left**) and Cross-Correlation (**right**).

Figure 10 shows boxplots that represent the distribution of different values used to measure similarity in the registration. As for this section, the nine rightmost boxplots are of interest; the boxplot labelled as dGW corresponds to our solution. The following four boxplots correspond to the original VM-2; the subindex indicated the type of training applied (see Section 4.2), while the term after the comma indicates the similarity metric (either Equation (11) or (5)). The last four boxplots show similar results for VMdiff [6]). In this case, we show one of the hyperparameters (λ); since this algorithm requires two, λ=10 is accompanied by the parameter σ=0.02 while λ=25 is accompanied by the parameter σ=0.01. As can be inferred from the figure, our algorithm shows a better performance than the VM and VMdiff alternatives. As for SSIM, significant differences exist for all the VM implementations $\left(p<10^{-7}\right)$.

**Figure 11:**
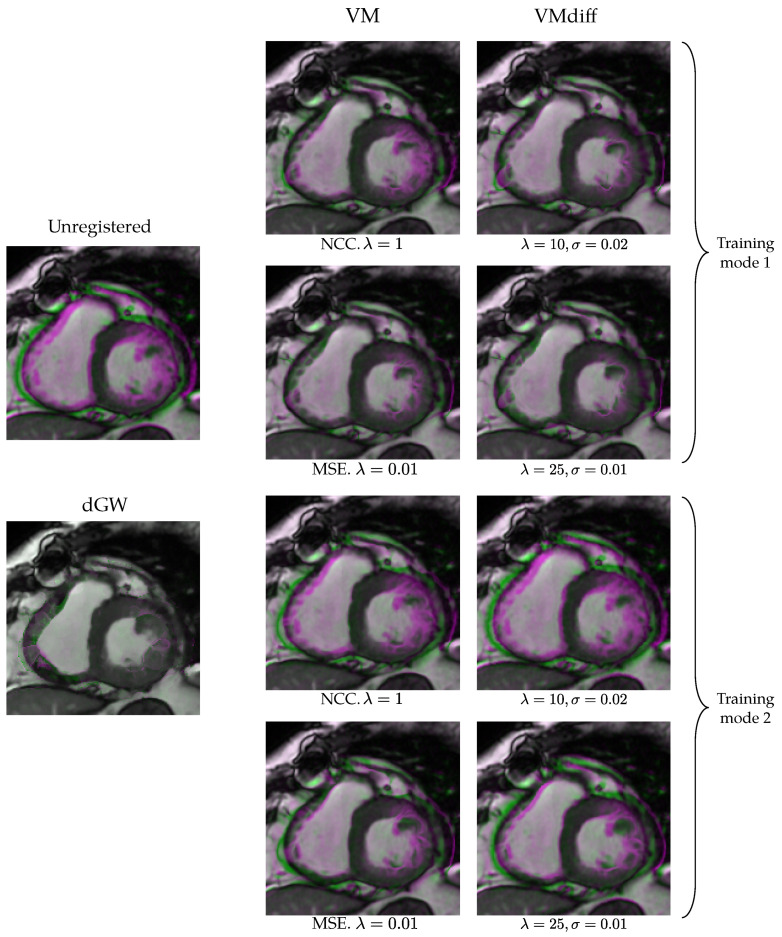
Differences between dGW and the eight VM implementation in registration quality. We show systole and a diastole superimposed frames for a slice of a patient in Database II. Leftmost column: unregistered cine cardiac MRI (up), dGW registration (down). Middle column: the four VM-2 implementations. Rightmost column: the four VMdiff implementations. Pink: registered systole with higher intensity. Green: registered diastole with higher intensity.

As an illustration, Figure 11 shows an example of registrations with dGW and the eight VM implementations. As indicated in Section 4.2, the presence of color indicates the presence of differences in the images. This example seems to corroborate the general trends shown in Figure 10.

### 5.3. Results of Experiment 3

The two rightmost boxplots in Figure 10 represent the distribution of the metrics used to measure registration quality for GW and dGW. The times taken in the registration process were 47.13±14.23 for the GW procedure while ours took 5.29±0.06 (both expressed in seconds and with the format average±estimated standard deviation).

Figure 4 shows the evolution of average SSD with the number of iterations in the algorithm for both GW and dGW and the test set in Database II. We have drawn a vertical line in L=5 to indicate the ordinary number of iterations that we use in dGW. For this experiment; however, we executed a higher number of iterations to check whether this higher number provides additional value; this does not seem to be the case.

**Figure 12:**
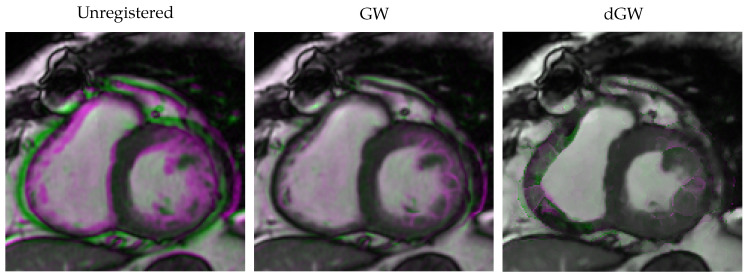
Differences between GW and dGW registration. From left to right: unregistered cine cardiac MRI, GW registration, dGW registration. Pink: registered systole with higher intensity. Green: registered diastole with higher intensity.

As an illustration, Figure 12 shows the same example as that in Figure 11; the left and right most figures (labelled as “Unregistered” and “dGW”) coincide with the images on the leftmost column in the latter figure. The image in the center is the result for GW registration. Differences in this case are more subtle than for the VM case, accordingly with the boxplot differences in Figure 10.

### 5.4. Results of Experiment 4

**Figure 13:**
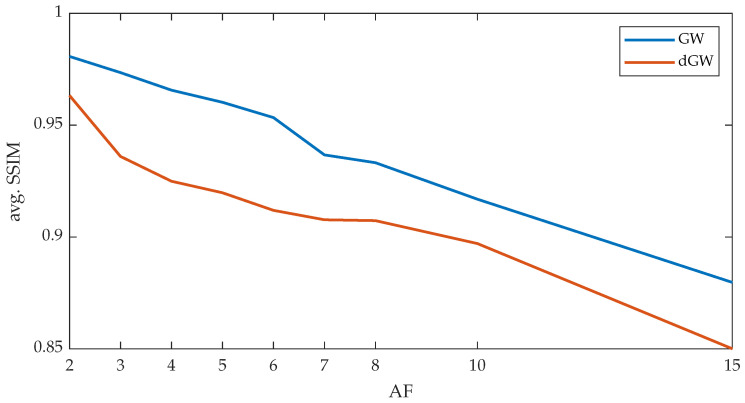
Average SSIM obtained, for different AF values, between the ME/MC reconstructions carried out with dGW and GW, with respect to the fully sampled reconstruction.

As a proof of concept, several reconstructions were performed for different AFs, using the dGW method and the GW method published in [7], for which a single iteration of ME/MC is carried out. Figure 13 shows the different average SSIM values for the sequence under study, when comparing a fully sampled reconstruction with the ME/MC reconstructions performed with dGW or GW.

**Figure 14:**
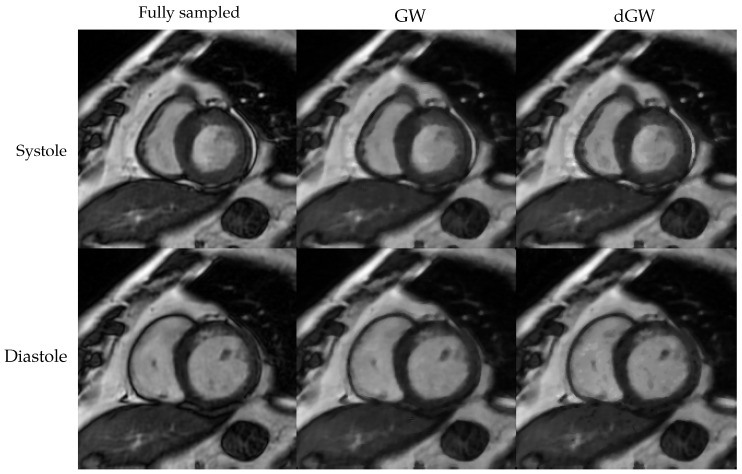
Systole and diastole reconstructions with AF = 8. Upper row: systole; lower row, diastole. Columns: from left to right, fully sampled reconstruction, GW and dGW.

Figure 14 shows the reconstructed images for AF = 8. To facilitate visualization, a frame corresponding to systole and another one corresponding to diastole are presented. As stated in the Introduction, our reconstruction results seem comparable to those obtained with the original method, while registration times are noticeably reduced. No clear differences between the two types of reconstruction are appraised.

## 6. Discussion

In this work, we propose a GW registration methodology based on DL techniques that can accommodate both monomodal and multimodal metrics. The registration consists of a network composed of a CNN and a spatial transformation; the network undergoes an unsupervised training for which there is no need for landmarks or any sort of labelling; self-similarity within the sequence with respect to the template has been pursued as a design criterion. This self-similarity can be defined for either monomodal or multimodal images. Results in Section 5.1 reveals that our method can deal with both types of metrics with satisfactory results; SSD turned out to work better than NCC but the latter was did not go through an hyperparameter optimization procedure, so room for improvement exists.

Our solution is image-based as opposed to patch-based [37,38]. The latter may need to include an adaptive sampling strategy to assure that a sufficient number of patches is used as well as to select the most representative patches for the entire training set [39]. This overhead is not necessary with our dGW.

We have adopted a methodology based on the forward selection method to avoid an exhaustive number of hyperparameter combinations. Despite this solution is known to be suboptimal, it is our understanding that this approach is worth taking as a balance between quality and manageability of the required training. We have observed that although most of the hyperparameters (recall Equations (3) and (10) as well as Figure 5) are significant in isolation, when tested in pairs no additional benefits are obtained with respect to using $\lambda_{3}$) as the unique non-null hyperparameter. This may be explained by the fact that the *Spatial transformation* block (recall Figure 1) carries out an implicit spatial regularization with the interpolation but no explicit provision is made in Equation (2) to foster time continuity in the deformation fields, i.e., to enforce time-smooth deformation fields. In addition, the constraint parameter $\lambda_{c}$ (recall Equation (10)) does not seem so relevant; the network may be learning some sort of periodicity through the time continuity and the periodicity of the time derivatives through $\lambda_{3}$ (specifically, notice that time derivatives in Equations (7) are calculated as “mod N” finite differences, so frame *N* makes use of the deformation fields at frame 1). Interestingly, as observed in Section 5.1, the network is robust to a random selection of the starting point in the cardiac cycle, but performance degrades with a shuffle in cardiac phases within the cardiac cycle. This seems coherent with the fact that shuffled cardiac phases would give rise to discontinuities in the motion fields.

Registration performance in terms of the four metrics shown in Figure 10 draws higher figures for our dGW (second leftmost boxplot) with respect to the four implementations of VM (next four boxplots) and the four implementations of VMdiff (four rightmost boxplots). *p*-values for the eight *t*-tests provided significative differences as well. We must say, however, that these two approaches are PW and not originally intended for dynamic sequence registration but for an atlas registration, for which the template role would be played by the atlas. This being the case, we have made an effort to adapt the training of these architectures to our case, as detailed in Section 4.2; this adaptation makes the network, in fact, behave as a GW solution since we have used a batch size that comprises all (or most) of the frames in the sequence. No iterations, however, are applied, nor the template is updated. These two shortcomings may explain the lower performance of these networks reflected in Figure 10. Unfortunately, no comparisons could be made with [4] because of three reasons; first, the paper, in our opinion, does not include sufficient detail to replicate the method. Second, the code is not available. Third, we meant to replicate their network architecture but, when it was launched, our GPU could not give room to the network, so training was prohibitively long. We stress that our approach, on the other side, runs smoothly on an average GPU in the market (recall the specific hardware used from Section 3.4.5).

dGW was also compared with GW, an optimization-based groupwise registration method used by our team for an MR reconstruction procedure. Figure 10 shows better performance from dGW, although differences in an actual image (as shown in Figure 12) turned out to not to be so remarkable.

Finally, reconstruction performance has provided comparable results in terms of visual inspection for GW and dGW; it should be noticed, however, that GW should be currently taken as an upper boundary for our performance since the network has not been so far trained with undersampled data. This has been set aside in this paper to focus on the canonical design of the network as a GW registration tool; this is, consequently, a current limitation of our solution. Approaches in which our solution is jointly trained within and end-to-end reconstruction system [20] seem worth exploring. Nevertheless, and as for registration runtimes, results in Section 5.3 reveals that dGW has a 9-fold average gain with respect to a traditional optimization-based solution; this is the objective we pursued. This paper is an extended and improved version of our conference paper [21]; in this improved version we have used a different network architecture, a different methodology to choose the template and a different loss function. In addition, hyperparameter selection has been here carried out based on a widely admitted procedure and experiments in this paper are more exhaustive, including a comparison with VM.

## 7. Conclusions and Future Work

This paper proposes a DL-based approach for GW elastic registration of a dynamic time sequence. An unsupervised approach has been used for training and our solution is image-based as opposed to patch-based. Although we have focused on a monomodal application domain, the solution can handle multimodal metrics easily.

We have provided an iterative solution and have observed that the iterations play a role in the registration quality. We have identified the network hyperparameters by a forward selection procedure and we have observed the network robustness with the image ordering within the sequence. In addition, we have compared our architecture with a state-of-the-art solution and results favored our design. Finally, as a proof of concept, we have obtained a MR reconstruction from undersampled data. Results were comparable with an optimization-based solution while registration runtimes were clearly reduced.

Our method is focused on registration; several applications can be devised different from image reconstruction; any application that requires material point tracking in a 2D dynamic sequence may benefit from our method. As for reconstruction, the current limitation is that training has been done with fully sampled data; training with undersampled data is now work in progress.
